# Role of an Exclusion Diet (Reduced Disaccharides, Saturated Fats, Emulsifiers, Red and Ultraprocessed Meats) in Maintaining the Remission of Chronic Inflammatory Bowel Diseases in Adults

**DOI:** 10.3390/medicina59020329

**Published:** 2023-02-09

**Authors:** Maria Nitescu, Doina Istratescu, Carmen Monica Preda, Teodora Ecaterina Manuc, Edouard Louis, Mircea Manuc, Tudor Stroie, Mihai Catrinoiu, Cristian George Tieranu, Larisa Emanuela Badea, Letitia Tugui, Adriana Andrei, Mihai Mircea Diculescu

**Affiliations:** 1 University of Medicine and Pharmacy “Carol Davila”, 050474 Bucharest, Romania; maria.nitescu@umfcd.ro (M.N.); carmenmonica.preda@gmail.com (C.M.P.); teodora.manuc@gmail.com (T.E.M.); m_manuc@yahoo.com (M.M.); stroie.tudor@gmail.com (T.S.); mihai.catrinoiu18@gmail.com (M.C.); mmdiculescu@yahoo.com (M.M.D.); 2 National Institute for Infectious Diseases “Prof. Dr. Matei Bals”, 021105 Bucharest, Romania; 3 Department of Gastroenterology, Clinic Fundeni Institute, 022328 Bucharest, Romania; larisabadea21@gmail.com (L.E.B.); letitiatugui@yahoo.com (L.T.); sandrei741@yahoo.com (A.A.); 4 Department of Gastroenterology, University Hospital CHU Liège, 4000 Liège, Belgium; edouard.louis@uliege.be; 5 Gastroenterology & Hepatology Department, Elias Emergency Hospital, 011461 Bucharest, Romania; tieranucristian@gmail.com

**Keywords:** inflammatory bowel disease, Crohn’s disease, ulcerative colitis, exclusion diet

## Abstract

*Background and Objectives*: Inflammatory bowel diseases are a main focus in current research, with diet being an emerging therapeutic line due to its links in both onset and progression. A Western-style diet high in processed foods, food additives, red meat, and animal fat has been linked to a higher risk of developing IBD. The aim of this study was to establish an association between an anti-inflammatory exclusion diet and maintenance of remission in IBD. Also, we assessed the efficacy and safety of this diet compared to a non-dietary group and the possible therapeutic effect of this diet in the maintenance of IBD remission. *Materials and Methods*: A total of 160 patients with IBD were screened for inclusion, but 21 did not met the inclusion criteria. Thus, 139 patients were assigned to either an exclusion diet or a regular diet according to their choice. *Results*: Clinical remission after six months was maintained in the exclusion diet arm (100%). In the control arm, four patients had clinically active disease (one patient with UC and three with CD), and 90 patients maintained the clinical remission state (95.7%) (*p*-value = 0.157). Regarding biochemical markers, ESR at baseline was higher in the exclusion diet arm: 29 (5–62) versus in the control arm 16 (4–48) (*p*-value = 0.019), but six months after, the groups were similar (*p*-value = 0.440). *Conclusions*: Patients who followed an exclusion diet maintained clinical remission more frequently. However, the threshold for statistical significance was not achieved. There was also a trend of improvement in inflammation tests in the intervention group.

## 1. Introduction

Inflammatory bowel diseases (IBDs) are a group of chronic idiopathic gastrointestinal afflictions, mainly represented by ulcerative colitis (UC) and Crohn’s disease (CD), that are characterized by exacerbation and remission periods [1,2,3,4]. They are related to significant morbidity, a lower quality of life and a growing financial burden on society due to direct and indirect expenses [5,6,7,8].

One of the environmental factors linked to the onset and progression of IBD is diet. Increasing data suggest that gut dysbiosis and an abnormal immune response occur in people who are genetically susceptible to them. This process is likely initiated and perpetuated by changes in environmental factors, including nutrition [1,9,10,11,12,13]. Numerous epidemiological studies indicate that a Mediterranean-style dietary pattern, which mostly consists of fiber-rich food sources, such as fruits and vegetables, and food sources high in omega-3 fatty acids, is related to a lower risk of developing IBD. On the other hand, a Western-style diet high in processed foods, food additives, red meat, and animal fat is linked to a higher risk of developing IBD [5,14,15,16,17,18,19].

A previous retrospective study involving two European cohorts (Eastern and Western Europe) conducted by our team demonstrated significant differences in dietary patterns of people with IBD versus the healthy population using a detailed questionnaire that thoroughly assessed the eating habits of each subject [12].

Some dietary components can strengthen the immunological and barrier functions of the body, protecting it from inflammation. Dietary nutrients have an impact on the activity of the gut microbiota in addition to the host immunity and intestinal barrier functions. The changed gut microbiota can then affect the host’s physiology. Additionally, it is known that inflammation alters the metabolism of host immune and non-immune cells, as well as the gut microbiota. Therefore, the host’s and/or the microbiota’s requirements for particular nutrients may change in IBD [15,18,20,21,22,23,24,25,26].

Due to the varied nature of IBD, it has been challenging to provide a single dietary recommendation for all IBD patients, and the sorts of food that are tolerated widely differ amongst patients [2,14,16,20,27,28,29].

Diet is one of the most important modifying factors leading to IBD pathogenesis. It has also been reported by patients as an important precipitating factor for IBD flares. There is a growing interest in how diet and nutrition can alter microbiota and modulate cytokine release, thus leading to a significantly lower inflammatory state. Despite the wide range of therapeutic alternatives, medication may not always achieve the expected outcome. Therefore, identifying a diet that can either diminish inflammation or help maintain remission can be pivotal [14,15,16].

Our previous published data showed that patients with IBD had a diet high in processed foods, sugar, food additives, red meat, and animal fat compared to healthy persons [12], which could lead to alterations in the gut flora and immunological dysfunction leading to flares, so our hypothesis was that a diet that would exclude these proinflammatory food and was enriched with fiber and high in omega-3 fatty acids could actually improve IBD-related symptoms and would improve chronic intestinal inflammation.

The aim of this study was to establish if there is an association between an anti-inflammatory exclusion diet and maintenance of remission in IBD. The efficacy and safety of this diet is addressed in comparison to a non-dietary group while discussing possible therapeutic effects of this diet in the maintenance of IBD remission.

## 2. Materials and Methods

### 2.1. Study Design and Patients

We conducted a prospective nonrandomized clinical trial study and enrolled patients that were in clinical remission followed in the Gastroenterology Outpatient Unit of Fundeni Clinical Institute from September 2021 to June 2022. Remission was defined according to CDAI score (under 150 points) for patients with Crohn’s disease or Mayo score (under 3 points) for patients with ulcerative colitis.

The inclusion criteria were age over 18 years old, diagnosis of IBD confirmed by endoscopic, radiologic, and histologic evaluation at least 6 months before entering the study, and stable IBD therapy, meaning no changes in treatment within the last 12 weeks before enrollment and clinical remission of the disease at inclusion. The exclusion criteria were the presence of active disease, patients with short bowel syndrome, other gastrointestinal diseases including cancer and infectious diseases, a preexisting dietary program and disagreement to participate in the study.

A total of 160 patients with IBD were screened for inclusion. Fifteen patients had active disease and one patient refused to enter the study. Among the 144 patients with IBD who agreed to participate in the study, after explanation of the dietary intervention, 1 patient felt the diet too restrictive in relation to their lifestyle, 3 were lost to follow-up and 1 was not included due to a worsening of bowel disease that required treatment modification. Thus, 139 patients fulfilled the inclusion criteria and were assigned to either an exclusion diet or a regular diet according to their choice (see Appendix A).

At the first visit, all patients underwent complete blood count and measurement of C-reactive protein (CRP) levels, ESR and fecal calprotectin. IBD activity was defined using the Mayo score and the CDAI score. All patients were also asked to complete a questionnaire regarding their diet preferences in the last year (Appendix A). This questionnaire was previously validated in a retrospective cohort study involving two populations—Eastern and Western Europe—and it was assessed based on the last 3 months’ dietary preferences [12]. The consumption of sweets and sweetened beverages, processed and high-fat meat, fried food, salt, shop-bought ice cream and mayonnaise was significantly higher in the entire IBD cohort than in the healthy control group, whereas seed, nut, and yoghurt consumption was lower. Based on the differences obtained, we developed a dietary plan for IBD patients (Table 1).

We assembled these food lists after carefully examining popular special IBD diets such as Mediterranean [17] and Low-FODMAP [21]. We preferred the food-list approach due to its simplicity and ease of use in day-to-day dietary planning for IBD patients. These lists were approved by our nutritionist and were aimed at limiting nutritional deficiencies frequently encountered in special IBD diets.

After initial assessment, all patients were assigned based on their choice to two different arms: one arm that adopted an exclusion diet for six months (45 patients) and the other arm that maintained their habitual diet (94 patients). Every patient received a consultation of 30–45 min to explain the assigned diet and answer questions. The dietary arm avoided the prohibited food for a period of 6 months. They were encouraged to consume products from the right food list and any other type of food that was not included in the prohibited food list was allowed. The quantity of food ingested was not specified and caloric intake was ascertained initially by our dietitian. There was no change in the patient’s usual treatment.

During the six-month intervention, all patients were monitored by a dedicated clinician for changes in their disease status and diet compliance. Five other patients were excluded during the study: three were lost to follow-up, one chose to retire because the dietary pattern was too difficult for her, and one developed hepatic abscess.

Firstly, we wanted to assess compliance through the elimination of prohibited foods (Table 1) and secondly, the switched intake towards fruits and vegetables, lean meat, cereals and olive oil.

From the nutritional point of view, we aimed at maintaining the same caloric intake and not necessarily increasing/lowering weight. We were interested in shifting calorie intake from the prohibited food list towards the right food list.

Initially, we had envisioned a daily food diary for the patients, which as time went on proved lackluster due to limited compliance. As such, we opted for a telemedical approach and patients were contacted every 2 weeks to assess dietary compliance for the intervention arm. At the end of the study, the diet compliance was assessed mostly in the outpatient clinic and using telemedicine where it was necessary. Anthropometric measures including weight and height were measured and BMI (weight (kilograms) divided by the squared height (meters)) was calculated at the study beginning and at the end. Every patient was asked about dietary changes at 6 months, but there were not reported differences.

After this period, every patient was clinically assessed and had another full blood count and CRP, ESR, and fecal calprotectin measurements.

### 2.2. Statistical Analysis

Data analysis was performed using statistical software SPSS (20.0 version from IBM Corporation, Armonk, NY, USA). Normality of data was examined using the Kolmogorov–Smirnov test. Quantitative variables with parametric distribution are summarized as means and standard deviation, while variables with nonparametric distribution are summarized as medians with minimum and maximum. We used for comparison the independent sample *t*-test for normally distributed data or Mann–Whitney U test for the abnormally distributed data. Categorical variables are summarized as percentages and compared using Fisher’s exact test. Two-sided hypothesis testing was used, with a *p*-value of less than 0.05 considered statistically significant.

The sample size needed was 38 to 57 patients for each group, considering a type I error of 5% (α = 0.05) and type II error of 20% (β = 0.20, power = 80%) and the primary outcome variable of expected difference between the intervention and control groups in mean IBD clinical score, for an odds ratio (OR) of 0.2 to 0.3 and a ratio of controls to cases of 2:1.

## 3. Results

### 3.1. Baseline Patient Characteristics

The baseline characteristics of the study population are reported in Table 2. More than half of the patients were male (56.8%) with a median age of 40 years, and half had normal BMI (49.6%), an advanced education diploma (59.1%), and earned an average income (44.6%). Most of them were nonsmokers (77.7%), half denied alcohol consumption (59%), and a quarter had had a disease-related surgical intervention (23%). Although the number of patients with UC was similar to CD patients, the repartition between study arms was not the same. A third of patients with UC chose the exclusion diet, while a quarter of patients with CD chose this arm. Considering patients with CD, most of them were diagnosed between 17 and 39 years old (71.4%), the localization was predominantly colonic (42.8%), most had an inflammatory pattern (46.7%), and a quarter had concomitant perianal disease (27.3%). Of the UC patients, about half of them had a left-side colitis extension (54.8%). The exclusion diet and control populations were similar, except for immunosuppressive treatment. Only 33.3% of patients in the exclusion diet group had an immunomodulator compared to 68.1% in the control group (*p*-value < 0.001) and the difference remained statistically significant for combo therapy as well (*p*-value < 0.001). Approximately 30% of patients used another therapy for their disease (5-ASA in monotherapy or in association with other treatment), but the groups were not significantly different.

According to the IBD type, there were some differences regarding appendectomy status and disease-related surgical interventions: 15.6% of CD patients had had an appendectomy compared to 3.2% of UC patients (*p*-value = 0.013) and 40.3% of CD patients had had disease-related surgical interventions compared to 1.6% of UC patients (*p*-value < 0.001).

### 3.2. IBD Population

#### 3.2.1. Dietary Habits

All patients had to answer a questionnaire regarding their current dietary habits (see Appendix A). The only variable that was statistically significant (*p*-value = 0.045) was the consumption of sweetened beverages: in the intervention arm, 15.6% of subjects drank more than 1 L of sweetened beverages, while in the control arm, 5.3% of subjects drank the same amount. The acceptance rate of the exclusion diet in our cohort was 32.4%. The nutritional status at the end of the study varied slightly within groups, and no statistically significant difference was recorded (*p*-value = 0.640).

#### 3.2.2. Disease Activity during Dietary Intervention

**Figure 1:**
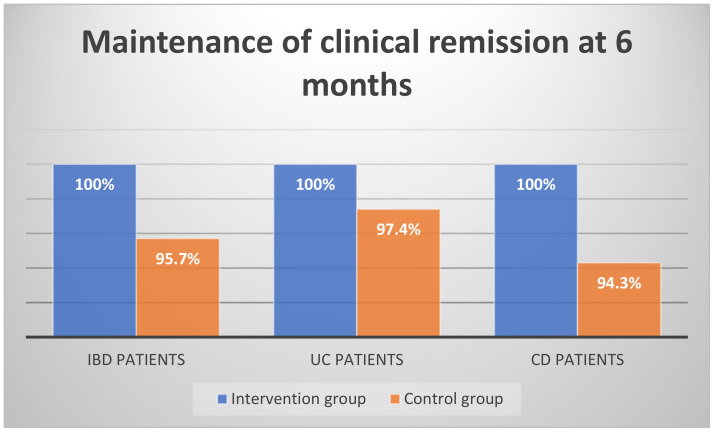
Maintenance of clinical remission 6 months after intervention.

Considering patients with CD, all of them were in remission at the beginning of the study. CDAI score at the beginning was 72.5 (5–148) for the intervention arm and 49 (−18–143) for the control arm (*p*-value = 0.183). CDAI score at the end of the study was 51 (−73–134) for the exclusion diet arm and 55 (−2–224) for the control arm (*p*-value = 0.224). As for UC patients, at the beginning of the study, Mayo score was 0 (0–2) for both arms (*p*-value = 0.641) and after six months Mayo score was 0 (0–2) for the intervention arm and 0 (0–7) for the control arm (*p*-value = 0.801). The clinical remission after six months was maintained in the exclusion diet arm (100%). In the control arm, four patients had active disease based on their clinical score (one patient with UC and three with CD) and 90 patients maintained the clinical remission state (95.7%) (*p*-value = 0.157) (Figure 1). The patients that had active disease at the end of the study were young (mean age 38) and did not report any other chronic treatment or lifestyle change during the 6-month period.

#### 3.2.3. Biochemical Markers of Disease Activity

Fecal calprotectin at baseline was higher than 300 micrograms/gram in 20% of cases in the exclusion diet arm and in 21.3% of cases in the control arm (*p*-value = 0.416). Subjects that had higher fecal calprotectin (>300 micrograms/gram) had a closer follow-up, and from those (29 patients), 17.3% had a treatment change, 44.8% had optimized therapy and 37.9% did not change therapy based on therapeutic drug monitoring (TDM), endoscopy or CT enterography. Six months later, 15.6% of cases had higher fecal calprotectin (>300 micrograms/gram) in the intervention arm and only 10.6% in the control arm (*p*-value = 0.067). Regarding ESR at baseline, we had a statistically significant difference between groups (*p*-value = 0.019): 29 (5–62) in the exclusion diet arm and 16 (4–48) in the control arm. At the 6-month checkpoint, the groups were similar (*p*-value = 0.440). Lastly, there was no statistically significant difference between groups regarding CRP, fibrinogen or hemoglobin at the beginning and 6 months after dietary intervention.

To take a deeper look into the results, we also performed a statistical analysis for each IBD type, maintaining the same groups: the exclusion diet arm and the control arm.

### 3.3. UC Subpopulation

For the UC subpopulation, appendectomy and surgical intervention status were not statistically significant between arms. In the exclusion diet arm, 34.7% of patients had had immunosuppressive treatment, whereas in the control arm, 71.8% of patients had had the same treatment (*p*-value = 0.009). Questioning the dietary habits, we obtained a statistically significant result for the consumption of sweetened beverages: 26.1% of patients included in the exclusion diet arm consumed more than 1 L per day versus 5.1% of patients included in the control arm (*p*-value = 0.018) (Table 3).

Regarding biochemical markers of the disease, 21.7% of patients in the intervention arm had a higher value of fecal calprotectin at baseline (more than 300 micrograms/gram) compared to 20.5% of patients in the control group (*p*-value = 0.910). Six months later, 21.7% of exclusion diet subjects maintained fecal calprotectin higher than 300 micrograms/gram compared to 10.3% of control subjects (*p*-value = 0.219). ESR was also statistically significant between groups (*p*-value = 0.001) at baseline, but not after six months (*p*-value = 0.267).

Clinical remission was maintained in 100% of exclusion diet cases and in 97.4% of control cases (*p*-value = 0.443) (Figure 1).

### 3.4. CD Subpopulation

In the CD subgroup, 31.8% of diet arm subjects had an immunosuppressive treatment compared to 65.5% of the control arm (*p*-value = 0.007) and 77.3% had had biologic therapy compared to 96.4% (*p*-value = 0.009). There was no difference regarding the consumption of sweetened beverages (Table 4).

Fecal calprotectin higher than 300 micrograms/gram at baseline in the diet arm was 18.2% and in the control arm 21.8% (*p*-value = 0.724). Six months later, 9.1% of diet patients had higher fecal calprotectin compared to 10.9% of control subjects (*p*-value = 0.814). There was no difference between groups regarding other biochemical values.

Clinical remission was maintained in 100% of diet cases compared to 94.3% of control cases (*p*-value = 0.258) (Figure 1).

## 4. Discussion

Diet plays an important role in the onset and progression of IBD. Although in recent years, the literature has become more interested in this field, there is currently no dietary approach that can be successfully applied to all IBD patients. This growing interest is also shared by patients that frequently ask what to eat and what to avoid. There is a lack of prospective interventional clinical trials with a longer duration of dietary intervention that could quantify the beneficial effects in IBD patients.

In this study, we aimed to assess the effect of an exclusion diet in the IBD clinical course. There is a previous observational study that compared two different European cohorts of IBD patients (Romanian and Belgian patients) that found significantly different dietary habits between IBD patients and control subjects regarding the consumption of sweets and sweetened beverages, processed and high-fat meat, fried food, salt, shop-bought ice cream and mayonnaise (higher consumption rate for IBD patients), and seed, nut and yogurt consumption (higher consumption rate for control group) [12]. Using this for an exclusion diet model, we decided to enroll IBD patients for assessing efficacy and adherence to diet in these patients.

Before entering the study, all patients responded to a dietary questionnaire and we observed that the proportion of patients that consumed sweetened beverages was significantly higher in the control arm, meaning that patients who chose to be part of the intervention group were more preoccupied by a healthy lifestyle and were more aware of the importance of diet in the evolution of IBD. Looking deeper into IBD subpopulations, in the UC subpopulation the significance was kept between the arms regarding sweetened beverage consumption. Racine et al. [30] in their study found a positive association between high consumption of sugar and soft drinks and UC development. Other studies concluded that low FODMAP diets improve functional gastrointestinal symptoms in quiescent IBD [20,31,32]. These studies support our findings and could be a reason why in the intervention arm there was no clinically active disease compared to the control arm.

Our study diet excluded red and processed meat. Reif et al. [33] found an association between the consumption of red and processed meat and the consecutive development of IBD. An abundant diet in red, processed meat increases the risk of relapse in UC (OR 5.19 (95% CI 2.1–12.9)) [34], but an exclusion diet does not reduce the rate of flares in CD [35]. A small prospective study of 22 CD patients in Japan found that following a semi-vegetarian diet, where only fish was allowed once a week and meat was allowed once every two weeks, was associated with remission in 94% of cases (15 of 16) compared to 33% of patients (2 of 6) in the omnivorous diet group [36]. The disparities between the available data could be the result of different study designs, including different diet intervention designs and regional eating habits (Asian versus Western eating habits).

We believe that the main reason for the low adherence to our exclusion diet (32.4%) was the exclusion of red and processed meat for a long period of time and the consumption of fatty fish two times a week. Romanian people have a long tradition of pork and beef consumption in winter and Easter, and it was difficult for the majority of them to abstain from this habit for 6 months. Fish consumption is scarce in our country, and only 15% of patients affirmed that they ate fish a minimum of 2 times a week on a regular basis.

The role of dairy products has been long debated in the literature. Dairy products are usually avoided by people with UC, since lactose has been linked to the condition [37]. In 1964, Truelove and Wright found no statistically significant difference between UC patients following a dairy-free diet and patients on a normal diet [38]. Another study with similar results for pediatric patients with UC was published by Strisciuglio et al. [39]. On the other hand, Eadala et al. [40] found a link between lactose sensitivity and IBD patients (both UC and CD patients). Another review by Mishkin found a better correlation between small bowel CD and lactose malabsorption than other types of IBD [41].

More than half of our population reported a consumption of high-lactose products daily, irrespective of study group. Our proposal was to exclude these products and to have them eat instead yogurt, low-fat cheese and lactose-free milk. Another reason for the low adherence to diet was the higher cost of those products and the low or no availability in rural or small urban areas.

A high-fiber diet lowers the likelihood of developing IBD; therefore, fiber consumption protects against the onset of IBD [32]. Ananthakrishnan et al. [42] affirmed that a fiber-rich diet may decrease CD risk by 40%. However, there is no protective effect against UC. Another study of 130 individuals under 30 years of age confirmed the role of dietary fiber consumption in the prevention of CD [43]. Using a high-fiber diet, our intervention arm had a clinical remission of 100% irrespective of IBD type, but we also excluded other food types associated with a high risk of IBD relapse.

According to Sakamoto et al. [44], there is a negative effect between the consumption of unsaturated fatty acids and the risk of IBD development. Unsaturated omega-3 fatty acid consumption has been shown to reduce the risk of developing UC [45]. Consuming linoleic acid, a polyunsaturated omega-6 fatty acid, has been shown to have an impact on the likelihood of developing UC. Arachidonic acid usage may also raise the incidence of UC, whereas an increase in the availability of the monounsaturated fatty acid oleic acid is a prophylactic measure [46]. Compared to oils made from seeds or fruits, extra-virgin olive oil is notably rich in phenolic components. These phenolic chemicals lessen the signs of chronic inflammation in IBD and help avoid oxidative damage to colon cells [47]. Consuming 50 mL of extra virgin olive oil reduced ESR (*p*-value = 0.03) and CRP (*p*-value = 0.001) compared to canola oil, according to a recent crossover study of 32 patients [48]. Our diet also excluded omega-6 rich oils, advising patients to include in their diet olive oil, resulting in a better maintenance of the remission rate in the intervention group.

The originality of this research consists of a diet based on our previous observational study, a study that compared two region’s cohorts (East versus Western Europe) and excluded the food categories that were statistically significant for IBD patients compared to controls [12].

Although there are multiple diets proposed for IBD patients, contradictory results have been found for a number of foods, and not all of a food’s effects on IBD have yet been established. In the exclusion diet that we proposed, patients with IBD were given a list of foods that have been demonstrated to be safe or helpful in IBD or should be avoided since they exhibit negative consequences. The main strength of the study is that it is a prospective clinical trial on 139 IBD patients. We have to take into account that there are few studies in the literature that focus only on the adult IBD population, as the majority were conducted on pediatric IBD subjects and extrapolated in clinical practice.

Another strength of our research was the duration period of the exclusion diet and follow-up (6 months), which we thought was a reasonable period of time to estimate long-term remission or a flare of the disease. Although a relatively small number of patients agreed to this diet, all of them managed to complete the 6-month period without difficulties, meaning that the exclusion diet can be tolerated for longer periods of time in order to help maintaining remission.

Of interest in our cohorts is the fact that only 33.3% of patients in the intervention arm benefited from immunosuppressive therapy compared to 68.1% of patients in the control arm (*p*-value < 0.001). As for biologic therapy, in the CD subpopulation we also had a significant difference (77.3% in the exclusion diet group versus 96.4% in the control group) (*p*-value = 0.009). This difference between the population groups could mean that the control arm had more tightly controlled disease, with a lower risk of flares during the study. As the populations were not similar regarding treatment, we could not help but wonder if this is the real reason why we could not obtain a statistically significant difference for clinical remission between groups.

A sensitive and accurate indicator of intestinal inflammation is fecal calprotectin. IBD has a very strong negative predictive value of a normal level. Contrarily, levels above the assay reference level (normally defined as 50 micrograms/gram stool) have a low positive predictive value. This increases the positive predictive value while only slightly lowering the negative predictive value [49,50,51]. In the STORI research, patients who stopped anti-TNF with mucosal healing and calprotectin levels above 300 micrograms/gram had a 30% relapse rate, compared to patients who experienced both mucosal healing and lower calprotectin levels, who experienced relapse rates of 10% to 20% [52,53].

We chose the same level of fecal calprotectin as the STORI study based on their reports. In our population, fecal calprotectin did not correlate with the clinical score at baseline or after intervention, no matter the arm of the study. This could be the result of a better-adjusted treatment for the disease in the control group, as there were 68.1% of patients on immunosuppressive therapy compared to only 33.3% in the intervention group.

Measuring fecal calprotectin merely once to anticipate relapse is not enough in everyday practice while monitoring a patient in clinical remission. In fact, the positive predictive value over one year or even longer periods of time was actually linked to shorter-term prediction in several longitudinal studies measuring fecal calprotectin at baseline. Thus, it is necessary to take serial measures of fecal calprotectin. The interpretation of such serial measures of calprotectin is still up for debate because there are so few data of this kind available [53,54,55,56].

The main limit of our study is the small number of patients included in the intervention arm. We had a very small acceptance rate (32.4%), despite the fact that it was not a very restrictive diet. Multiple reasons were given by our patients: the difficulty of giving up on red meat and high lactose products for 6 months, the higher cost of a diet based on low-fat cheese, lactose-free milk, whole-grain bread and cereals, olive oil, the unavailability of certain products in the local markets, and a lack of information regarding the role of diet in IBD (there were patients that did not see its utility since they were already in remission).

There are several studies in the literature that quantified the dietary beliefs of the IBD patients. Avoiding certain foods was the most common behavior to lessen sickness symptoms, followed by consuming more of certain foods and then adhering to strict diets. This indicates that patients may prefer not to adhere to rigorous diets, yet avoid doing so by avoiding foods that are inappropriate or eating more meals that are considered advantageous [57,58]. These results could also explain the low adherence of the IBD population to our diet.

Another limit is the fact that during the study, we did not take into account the therapy changing, so we could not report if there are patients that benefit from another therapy at the end of the study.

## 5. Conclusions

In conclusion, we found a better trend of maintaining clinical remission in patients who followed the exclusion diet, but statistical significance was not obtained, probably due to the inhomogeneity of the groups. There was also a trend of improvement in inflammation tests in the intervention group, but not in fecal calprotectin. This strengthens our argument that IBD is a lifestyle-related disease caused by a Westernized diet. The exclusion diet is a well-tolerated diet (only one withdrawal), associated with higher rates of long-term clinical remission. These results are both positive and promising, and need to be confirmed in larger, randomized, controlled clinical trials.

## Figures and Tables

**Table 1:** The exclusion diet for IBD in the intervention arm.

Prohibited Food				
Red Meat	Ultraprocessed Food	Fried Foods	High-Lactose Foods	Others
Veal, pork, lamb Sausages	All type of processed meat Ice cream (bought from shop) Mayonnaise or bought from shop dressings Chips/nachos/other snacks Margarine Fruit yogurt (bought from shop) Pastry and cakes	Fried potatoes Fried meat Fried fish Fried vegetables Schnitzel and meatballs	Milk: cow, goat, sheep, ice cream Cheeses: fresh cheese (ricotta, cottage)	Fast food (burgers, pizza, shawarma) not allowed because it is ultraprocessed food and it contains high amounts of fat, emulsifiers and fried food White bread, soft drinks sweetened with sugar Vegetables oils rich in omega-6 (safflower oil, corn oil)
**The Right Food**				
**Fruits: 2–3 Servings/Day**	**Vegetables: 3–5 Servings/Day**	**Dairy Products**	**Cereals**	**Others**
Especially: banana, apple, grapefruit, currants, grapes, melon, kiwi, lemon, lime, tangerines, oranges, passion fruit, raspberries	Especially: carrots, celery, bell peppers, corn, eggplants without skin, zucchini, green beans, salad, chives, parsnips, pumpkin, beets, green onions (leaves), tomatoes, skinned pulses long term cooked	Lactose-free milk, rice milk Cheeses: only light cheese (12–14% fat) Natural yogurt Homemade ice cream, especially sorbet	Whole wheat bread, pasta Brown rice Barley Oatmeal	Lean meat: white meat, white fish (3–4 servings/week), fatty fish for omega-3 (2 servings/week) Olive oil Sweeteners: sugar (sucrose) max 10 g/day or glucose max 10 g/day, or honey max 10 g/day, or sweeteners (stevia) Psillyum 4 g/zi

**Table 2:** Baseline patient characteristics.

Parameter	All Patients (n = 139)	Exclusion Diet (n = 45)	Control (n = 94)	*p*-Value *
**Sex (male)**	79 (56.8)	22 (48.9)	57 (60.6)	0.205
**Age (years)**	40 (18–77)	44 (22–77)	38 (18–75)	0.221
**BMI**				
Underweight:	7 (5)	2 (4.4)	5 (5.3)	0.973
Normal:	69 (49.6)	23 (51.1)	46 (48.9)	
Overweight:	45 (32.4)	14 (31.1)	31 (33)	
Obesity:	18 (13)	6 (13.4)	12 (12.8)	
Education level				
0: less than basic education	0: 2 (1.4)	0: 0 (0)	0: 2 (2.1)	0.177
1: basic education	1: 12 (8.6)	1: 3(6.7)	1: 9 (9.6)	
2: intermediate education	2: 43 (30.9)	2: 12 (26.7)	2: 31 (33)	
3: advanced education	3: 82 (59.1)	3: 30 (66.6)	3: 52 (55.3)	
**Income**				
0: no income	0: 10 (7.2)	0: 3 (6.7)	0: 7 (7.5)	0.905
1: minimum wage	1: 12 (8.6)	1: 2 (4.4)	1: 10 (10.6)	
2: average wage	2: 62 (44.6)	2: 24 (53.3)	2: 38 (40.4)	
3: more than average wage	3: 55 (39.6)	3: 16(35.6)	3: 39 (41.5)	
**Non-smokers**	108 (77.7)	36 (80)	72 (76.6)	0.828
**No alcohol consumption**	82 (59)	30 (66.7)	52 (55.3)	0.269
**Appendectomy**	14 (10.1)	6 (13.3)	8 (8.5)	0.467
**Surgical intervention**	32 (23)	8(17.8)	24 (25.5)	0.342
**Disease type**				0.362
Ulcerative colitis	62 (44.6)	23 (37.1)	39 (62.9)	
Crohn’s disease	77 (55.4)	22 (28.6)	55 (71.4)	
**CD age at onset**				0.143
A1: <17 years	2 (2.6)	0 (0)	2 (3.6)	
A2: 17–39 years	55 (71.4)	14 (63.6)	41 (74.6)	
A3: >39 years	20 (26)	8 (36.4)	12 (21.8)	
**CD disease localization**				0.802
L1: terminal ileum	21 (27.3)	7 (31.8)	14 (25.4)	
L2: colon	33 (42.8)	8 (36.4)	25 (45.5)	
L3: ileocolon	18 (23.4)	6 (27.3)	12 (21.8)	
L4: upper GI	5 (6.5)	1 (4.5)	4 (7.3)	
**CD behavior**				0.808
B1: nonconstricting/nonpenetrating	36 (46.7)	11 (50)	25 (45.4)	
B2: constricting	20 (26)	5 (22.7)	15 (27.3)	
B3: penetrating	21 (27.3)	6 (27.3)	15 (27.3)	
+p: perianal disease modifier	21 (27.3)	8 (36.4)	13 (23.6)	0.271
**UC extent**				0.974
E1: proctitis	10 (16.1)	4 (17.4)	6 (15.4)	
E2: left-side colitis	34 (54.8)	12 (52.2)	22 (56.4)	
E3: pancolitis	18 (29.1)	7 (30.4)	11 (28.2)	
**Immunosuppressive treatment**	79 (56.9)	15 (33.3)	64 (68.1)	<0.001
Azathioprine	76 (54.7)	14 (31.1)	62 (66)	
Methotrexate	3 (2.2)	1 (2.2)	2 (2.1)	
**Biologic treatment**	114 (82)	33 (73.3)	81 (86.2)	0.097
Infliximab	42 (36.8)	11 (33.3)	31 (38.3)	
Adalimumab	35 (30.7)	12 (36.4)	23 (28.4)	
Vedolizumab	26 (22.8)	7 (21.2)	19 (23.5)	
Ustekinumab	8 (7)	2 (6.1)	6 (7.4)	
Tofacitinib	2 (1.8)	1 (3)	1 (1.2)	
Upadacitinib	1 (0.9)	0 (0)	1 (1.2)	
**Combo therapy**	71 (51.1)	13 (28.9)	58 (61.7)	<0.001
Other IBD treatment	44 (31.7)	19 (42.2)	25 (26.6)	0.080

* *p*-value is calculated for the difference between the group that received the exclusion diet and the control group.

**Table 3:** Baseline characteristics for the UC subpopulation.

Parameter	Exclusion Diet (37.1%)	Control (62.9%)	*p*-Value *
Gender	M: 60.9%	M: 61.5%	1.000
Age	Median: 44 (23–77)	Median: 43 (18–72)	0.662
**Immunosuppressive treatment**	34.7%	71.8%	0.009
Biologic treatment	69.6%	71.8%	0.853
**Sweetened beverages**	26.1%	5.1%	0.018

* *p*-value is calculated for the difference between the group that received the exclusion diet and the control group.

**Table 4:** Baseline characteristics for the CD subpopulation.

Parameter	Exclusion Diet (28.6%)	Control (71.4%)	*p*-Value *
Gender	M: 36.4%	M: 60%	0.079
Age	Median: 42.5 (22–72)	Median: 36 (20–75)	0.394
**Immunosuppressive treatment**	31.8%	65.5%	0.007
**Biologic treatment**	77.3%	96.4%	0.009
Sweetened beverages	4.5%	5.5%	0.872

* *p*-value is calculated for the difference between the group that received the exclusion diet and the control group.

**Figure A1:**
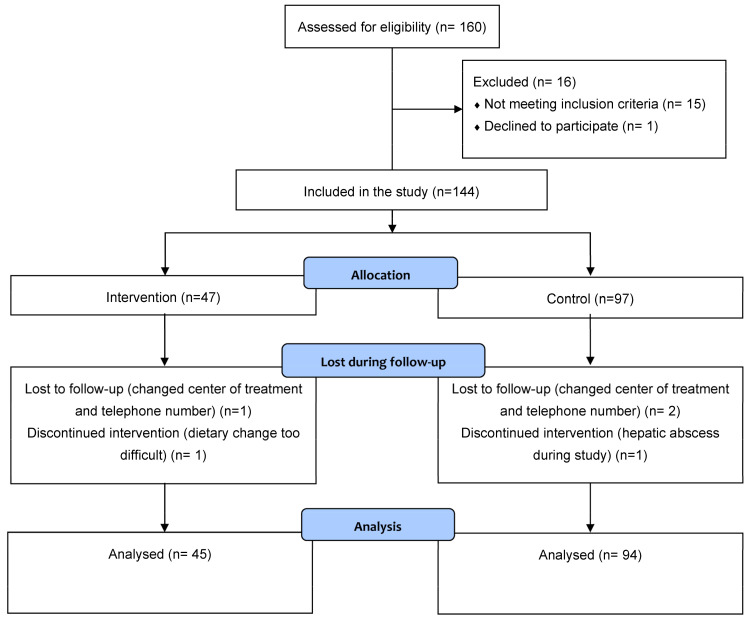
Study design.

## Data Availability

Not applicable.

## Appendix A

**Table A1:** Diet preferences questionnaire.

Food Group	Consumption
Vegetables >4 portions/day	Yes/no
Fatty fish—2 portions/week	Yes/no
Salt low/high	Yes/no
Sweetened beverages >1 L/day	Yes/no
Whole grains >3 portions/day	Yes/no
Seeds >4 portions/week	Yes/no
Processed meat >2 portions/week	Yes/no
Cheese daily	Yes/no
Natural yogurt daily	Yes/no
Fruit yogurt daily	Yes/no
Butter daily	Yes/no
Fatty meat daily	Yes/no
Margarine daily	Yes/no
Fried food daily	Yes/no
Ice cream daily	Yes/no
Mayonnaise daily	Yes/no
Chips daily	Yes/no
Coffee consumption	Yes/no
